# Supplementary material for: A generative adversarial network model alternative to animal studies for clinical pathology assessment
Source: Nat Commun. 2023 Nov 6;14:7141. doi: 10.1038/s41467-023-42933-9 (PMC10628291; doi:10.1038/s41467-023-42933-9)
Supplement: Supplementary file 1 — Supplementary Information [file 41467_2023_42933_MOESM1_ESM.pdf]

Supplementary Information for

A Generative Adversarial Network Model Alternative to Animal Studies for Clinical Pathology Assessment

Xi Chen[1], Ruth Roberts[2,3], Zhichao Liu[1,4*], Weida Tong[1*]

[1] National Center for Toxicological Research, Food and Drug Administration, Jefferson, Arkansas 72079, USA

[2] ApconiX Ltd, Alderley Park, Alderley Edge SK10 4TG, UK

[3] University of Birmingham, Edgbaston, Birmingham B15 2TT, UK

[4] Currently working at Integrative Toxicology, Nonclinical Drug Safety, Boehringer Ingelheim Pharmaceuticals, Inc., Ridgefield, Connecticut 06877, USA

*Correspondences: Weida Tong Weida.Tong@fda.hhs.gov and Zhichao Liu zhichao.liu@boehringer-ingelheim.com

Disclaimer

This manuscript reflects the views of the authors and does not necessarily reflect those of the Food and Drug Administration. Any mention of commercial products is for clarification only and is not intended as approval, endorsement, or recommendation.

Supplementary Figures

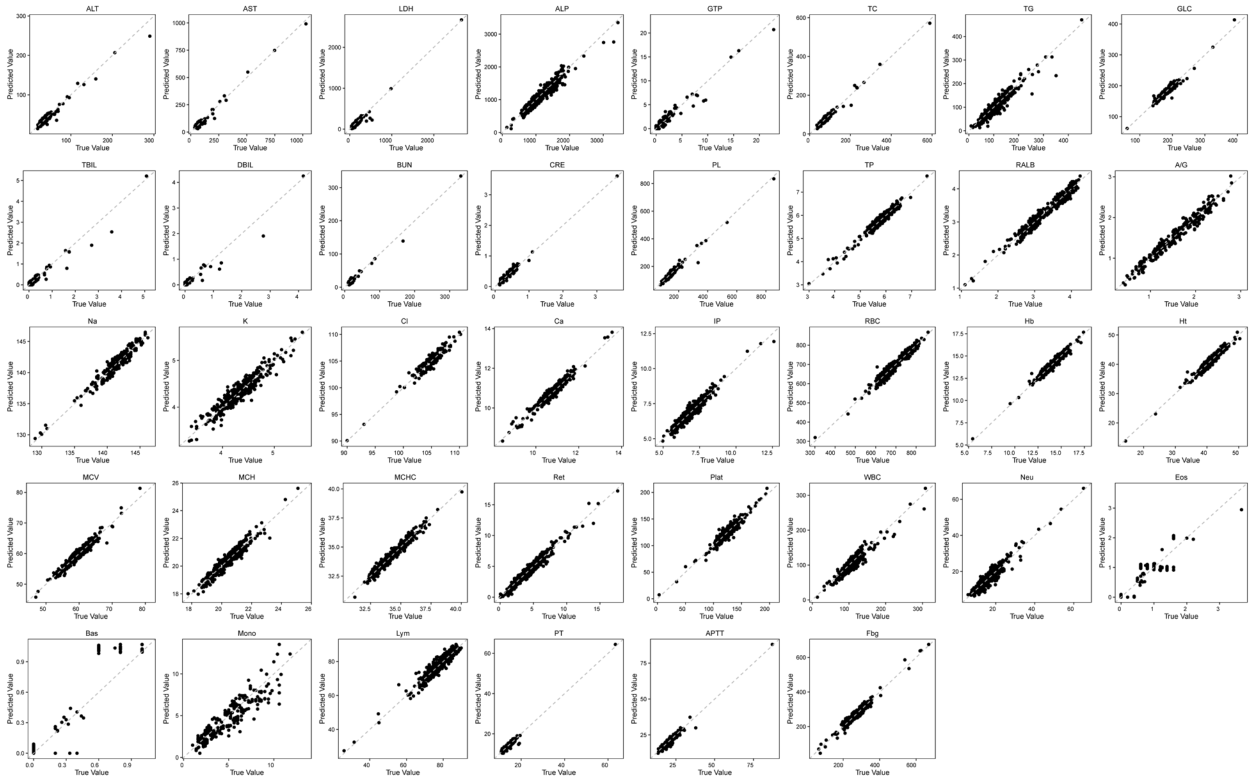

Supplementary Figure 1. Prediction error plots of AnimalGAN synthetic results against actual laboratory animal testing data for each of the 38 clinical pathology measurements. Each point represents a treatment condition in the test set. Points on the diagonal depict perfect prediction. Points below the diagonal were underestimated by AnimalGAN (predicted value is lower than the true value), while points above the diagonal were overestimated (predicted value is higher than the true value).

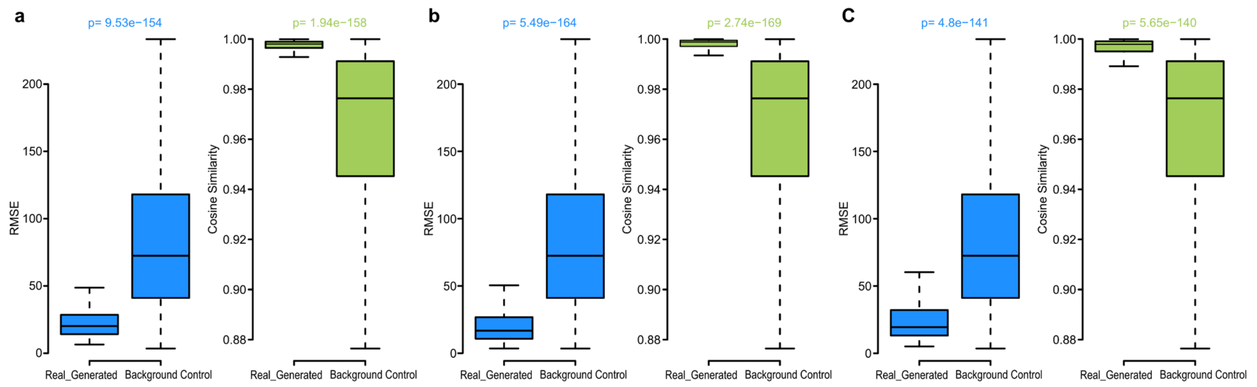

Supplementary Figure 2. AnimalGAN evaluation in three distinct real-world scenarios. RMSEs and Cosine Similarities between synthetic data and laboratory animal testing data for treatment conditions in test set comparing with those of background distribution. **a** Keep compounds whose chemical structures were far different from those that were used to develop AnimalGAN model in test set; **b** Keep drugs whose therapeutical classes were not included in the development of AnimalGAN in test set; **c** Keep drugs that were approved by FDA more recently in test set. The statistical difference between RMSEs/Cosine Similarities of AnimalGAN generated synthetic data and real animal testing data for treatment conditions in the test set (**a**, n=326; **b**, n=336 ; **c**, n= 333) and RMSEs/Cosine Similarities of real data across any two treatment conditions (n=1,358,776, derived from 1649×1648/2) was determined using a two-tailed Wilcoxon rank-sum test without adjustments for multiple comparisons. The boxplot displays the distribution of RMSEs/Cosine Similarities, with the centerline representing the median, the bounds of the box representing the first and third quantiles, and the whiskers representing the 1.5 times the interquartile range (IQR).

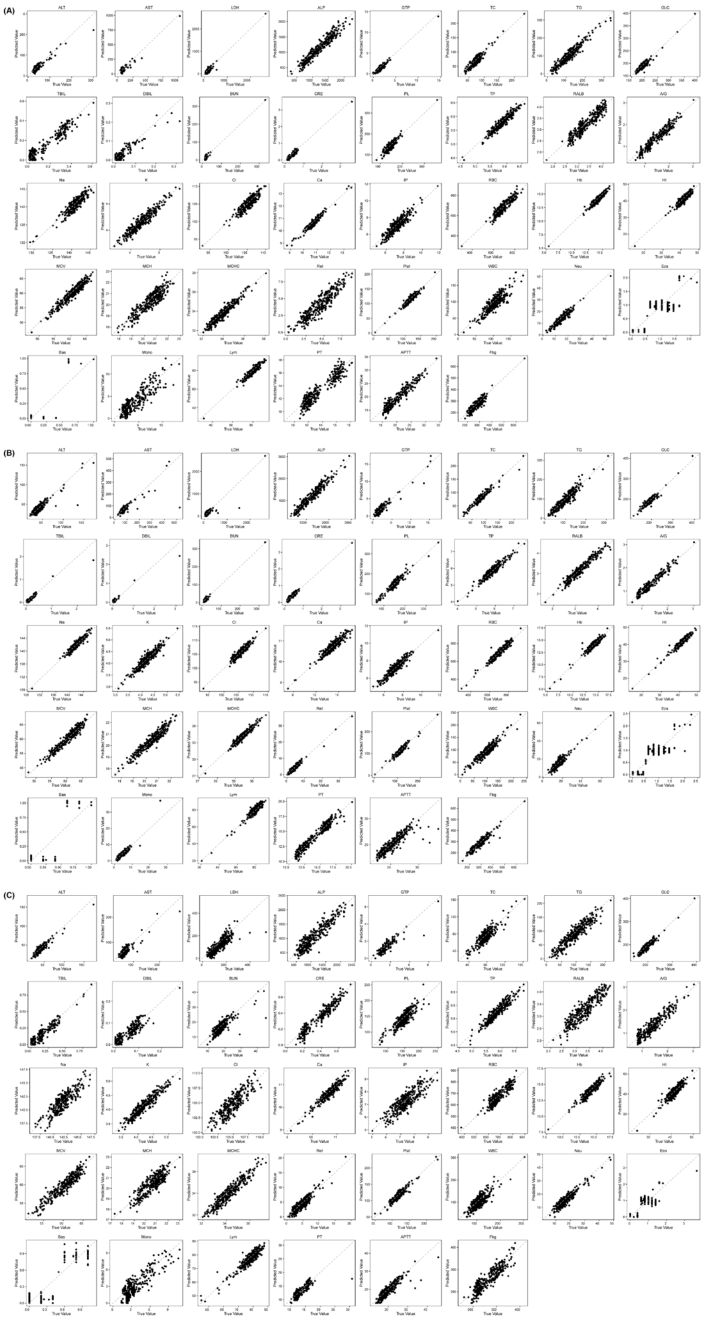

Supplementary Figure 3. Prediction error plots of synthetic results against actual testing values for each of the 38 clinical pathology measurements for treatment conditions in test set. (A) Keep compounds whose chemical structures were far different from those that were used to develop AnimalGAN model in test set; (B) Keep drugs whose therapeutical classes were not included in the development of AnimalGAN in test set; (C) Keep drugs that were approved by FDA more recently in test set. Each point represents a treatment condition in test set. Points on the diagonal depict perfect prediction. Points below the diagonal were underestimated (predicted value is lower than the true value), while points above the diagonal were overestimated (predicted value is higher than the true value).

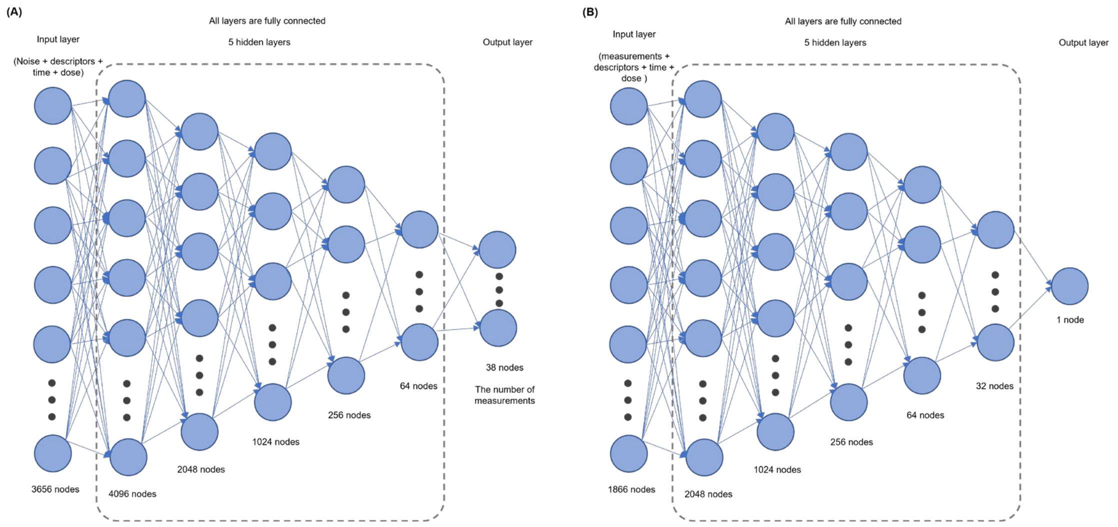

Supplementary Figure 4. Graphic representation of the AnimalGAN. (A) Generator. (B) Discriminator.

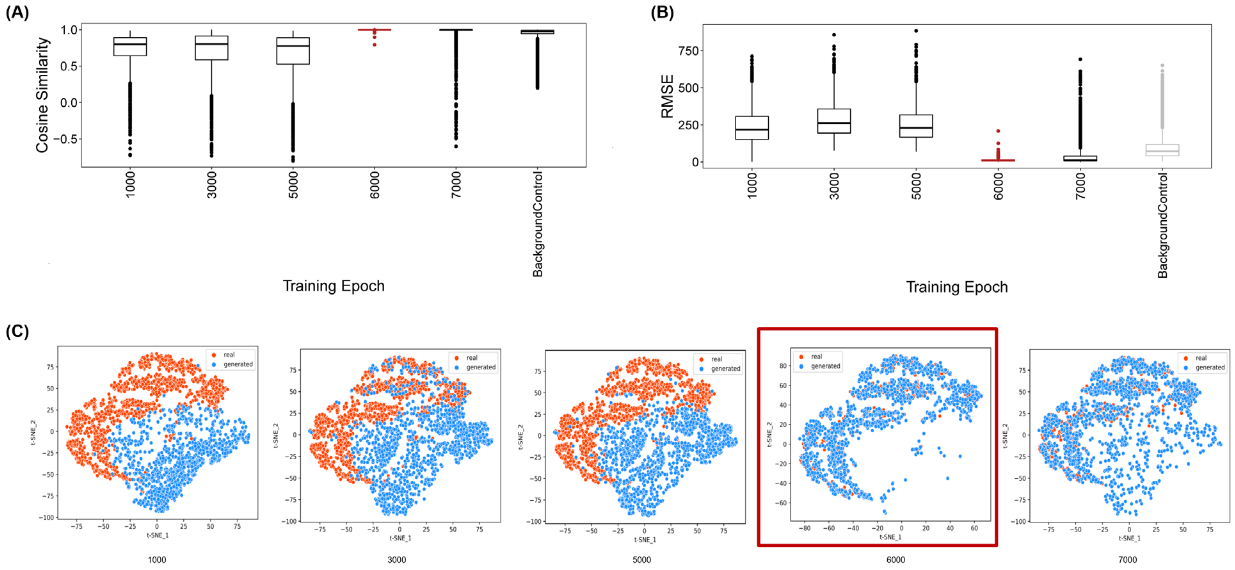

Supplementary Figure 5. Evolution of model performance during training. Distributions of (A) cosine similarities and (B) RMSEs between generated data and real animal testing data for all the treatment conditions in training set along with training. (C) t-SNE visualization of generated data and real data for treatment conditions in the training set at different training epochs. Each point depicted one treatment condition.

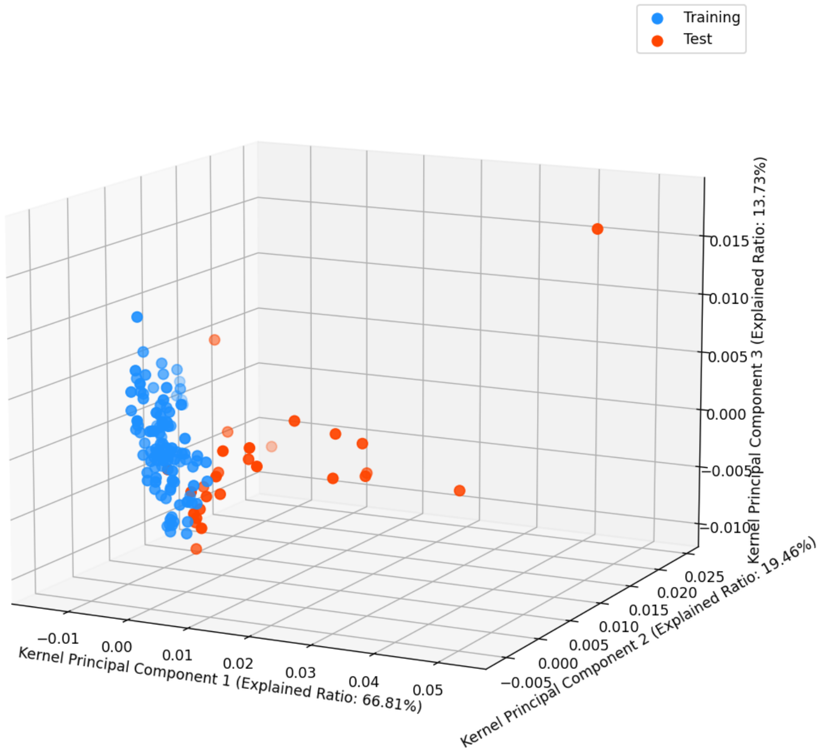

Supplementary Figure 6. Visualization of structural similarities of all the 138 compounds. The pairwise structural similarities between any two of the 138 compounds were calculated based on their Mordred molecular representations. Each point depicted one compound.

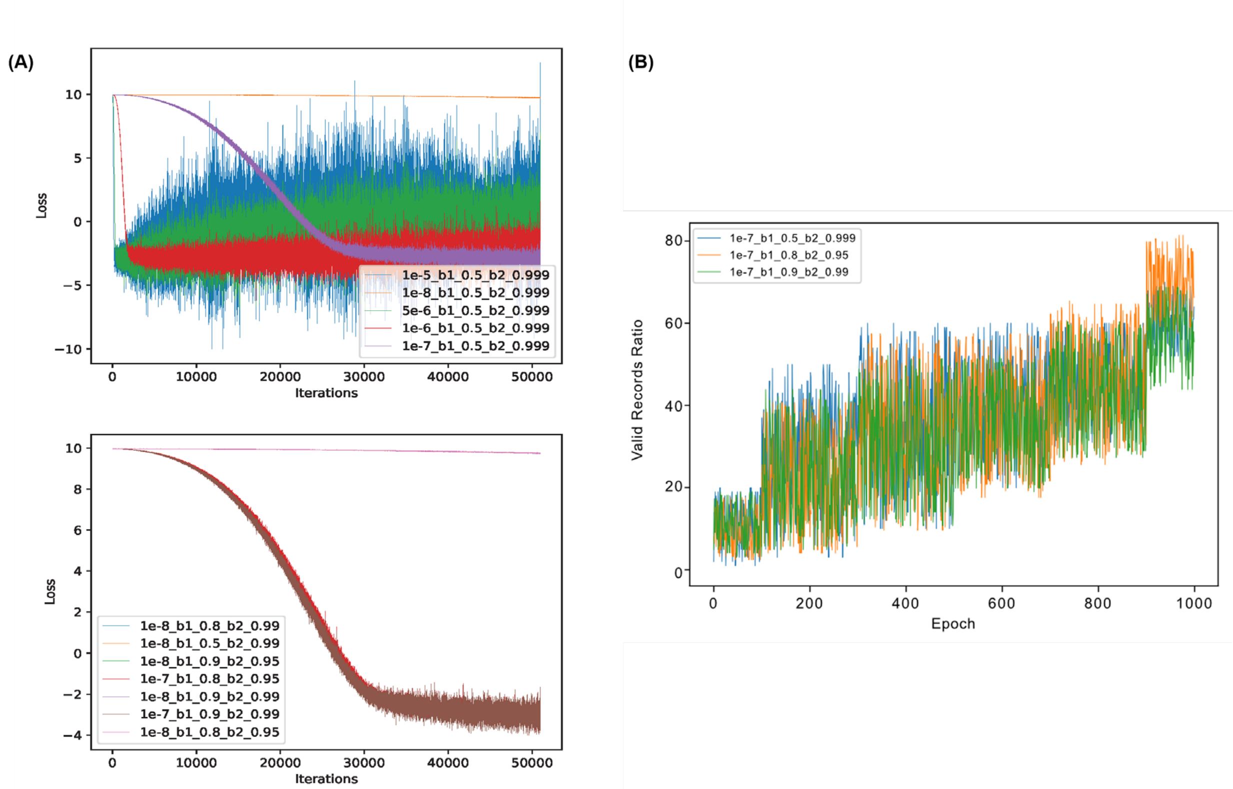

Supplementary Figure 7. Comparison for different learning rates when other parameters are fixed. (A) Loss curves of discriminators under different initial learning rates, b1, and b2 values. (B) Valid records ratios of all generated records under different initial learning rate, b1, and b2 values.

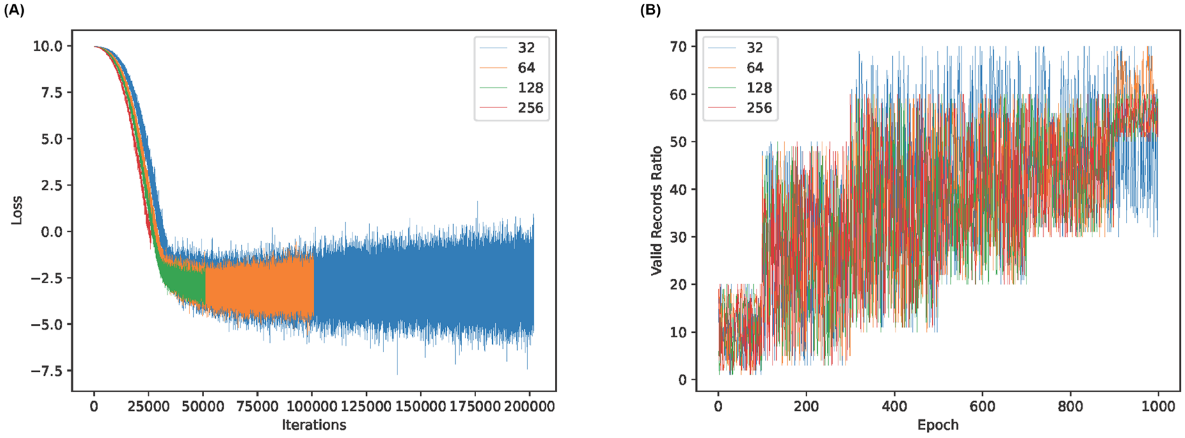

Supplementary Figure 8. Comparison for different batch sizes when other parameters are fixed. (A) Loss curves of discriminators under different batch sizes. (B) Valid records ratios of all generated records under different batch sizes.

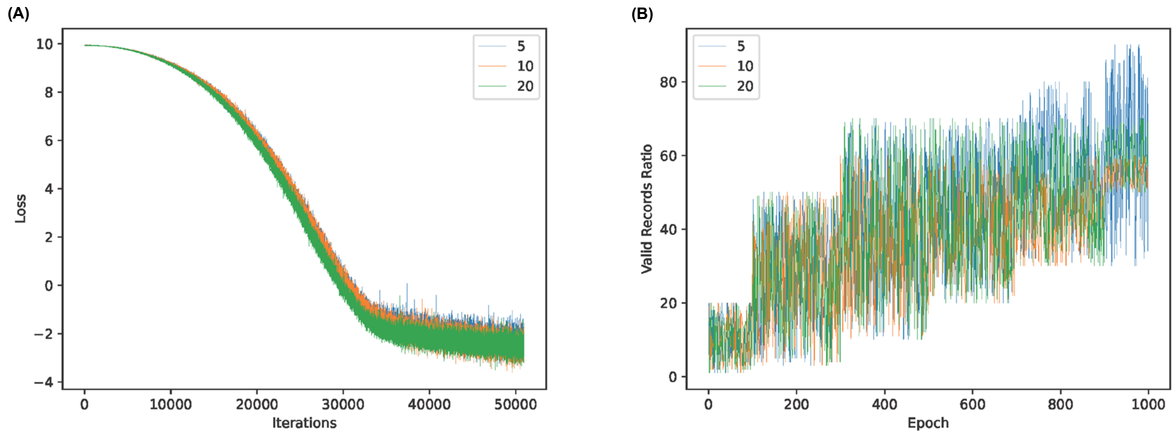

Supplementary Figure 9. Comparison for different numbers of discriminator iterations when other parameters are fixed. (A) Loss curves of discriminators under different numbers of discriminator iterations. (B) Valid records ratios of all generated records under different numbers of discriminator iterations.

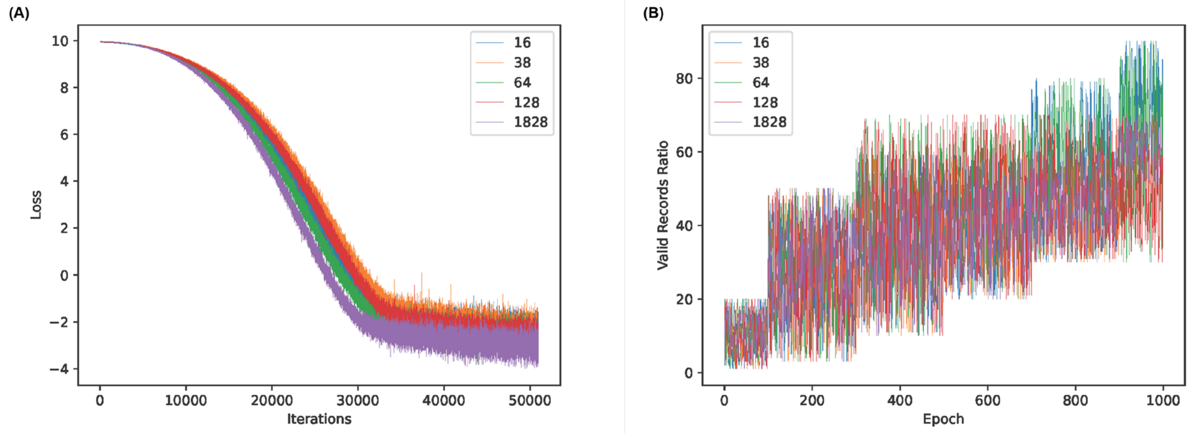

Supplementary Figure 10. Comparison for different noise length when other parameters are fixed. (A) Loss curves of discriminators under different noise length. (B) Valid records ratios of all generated records under different noise length.

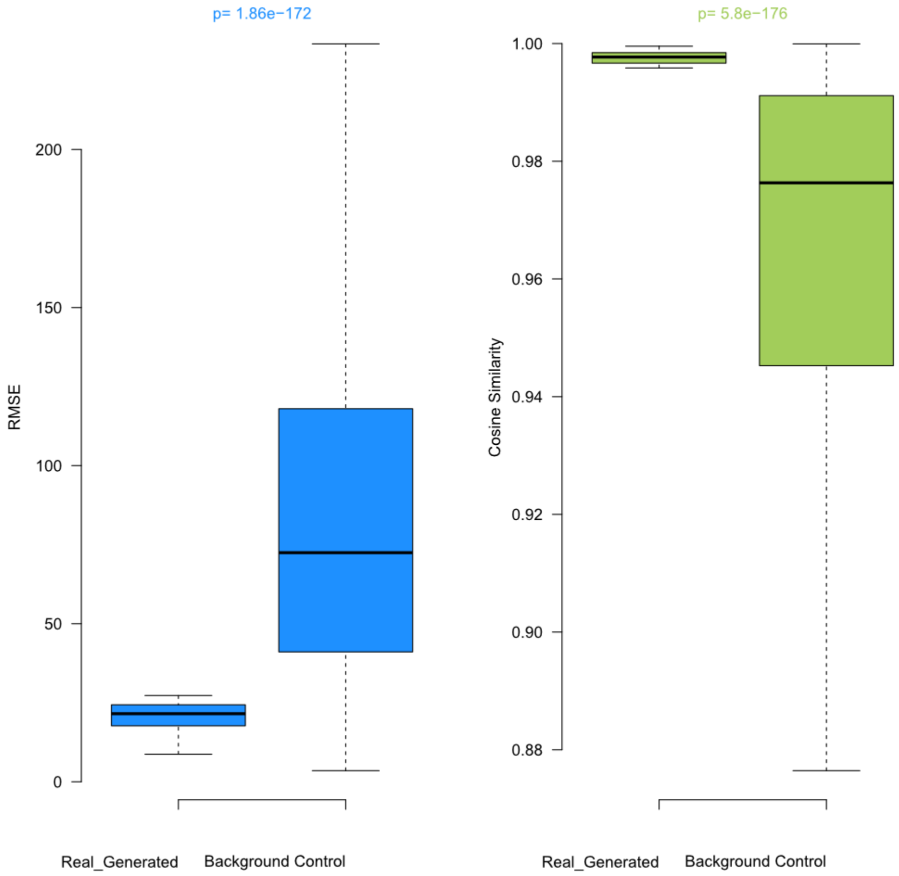

Supplementary Figure 11. Boxplot of RMSE - Root Mean Square Error and Cosine Similarity between generated synthetic data and real animal testing data for treatment conditions in the test set. The statistical difference between RMSEs/Cosine Similarities of AnimalGAN generated synthetic data and real animal testing data for n=332 treatment conditions in the test set and RMSEs/Cosine Similarities of real data across any two treatment conditions (n=1,358,776, derived from 1649×1648/2) was determined using a two-tailed Wilcoxon rank-sum test without adjustments for multiple comparisons. The boxplot displays the distribution of RMSEs/Cosine Similarities, with the centerline representing the median, the bounds of the box representing the first and third quantiles, and the whiskers representing the 1.5 times the interquartile range (IQR).

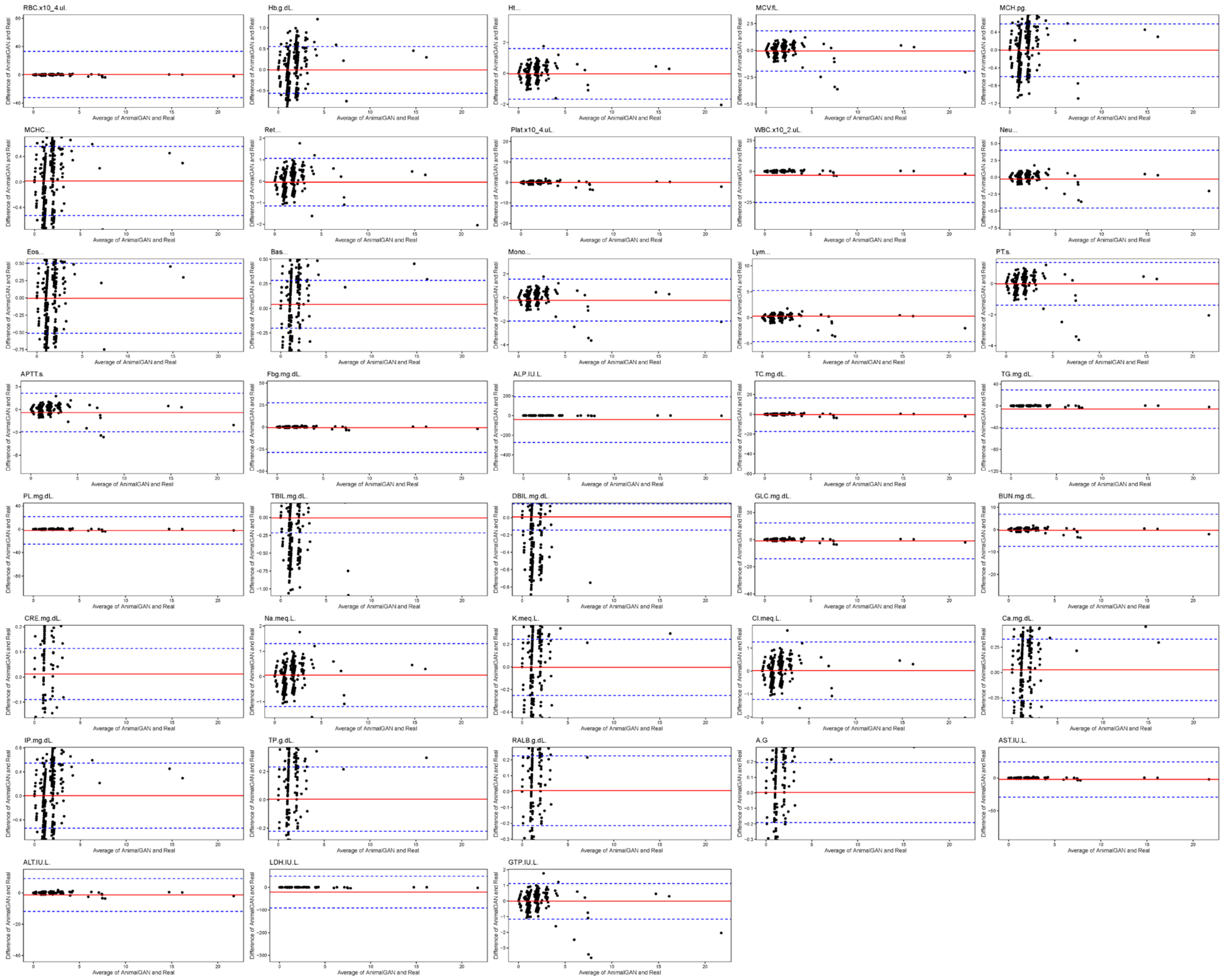

Supplementary Figure 12. Bland Altman analysis on 38 measurements.

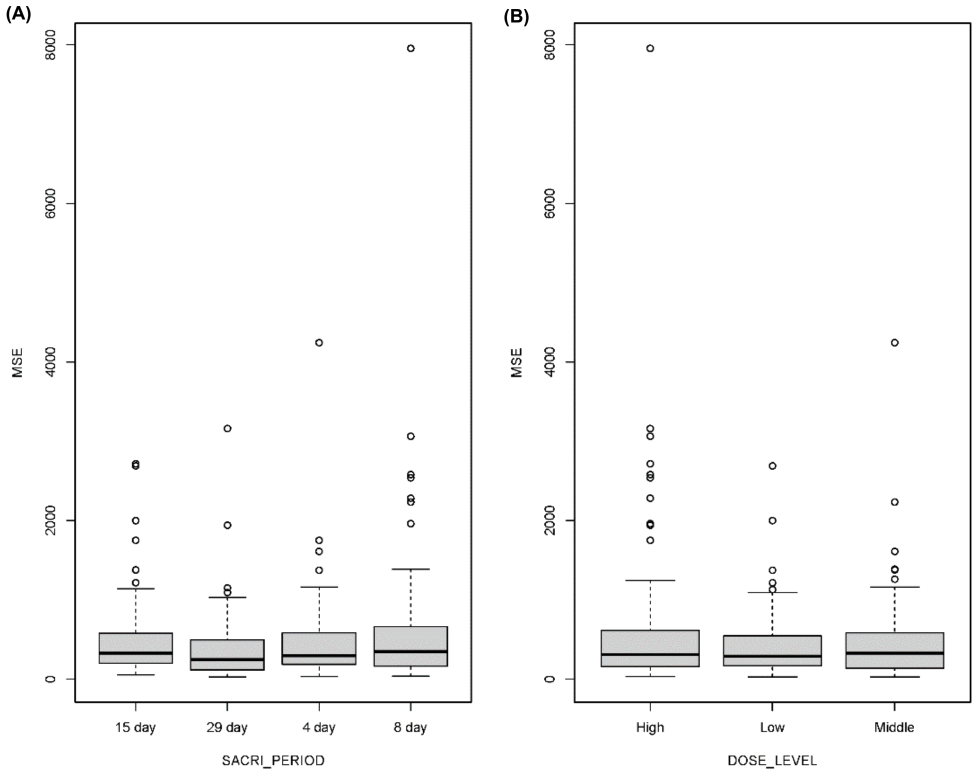

Supplementary Figure 13. MSEs over (A) time points and (B) dose levels.

Supplementary Tables

Measurement	Consistency	Toxicity
GTP	0.9608	Hepatotoxicity
LDH	0.9789	Hepatotoxicity
TBIL	0.9819	Hepatotoxicity
DBIL	0.9849	Hepatotoxicity
ALT	0.9910	Hepatotoxicity
AST	0.9970	Hepatotoxicity
ALP	1.0000	Hepatotoxicity
BUN	0.9789	Nephrotoxicity
K	0.9880	Nephrotoxicity
CRE	0.9940	Nephrotoxicity
Na	1.0000	Nephrotoxicity
Cl	1.0000	Nephrotoxicity
Ca	1.0000	Nephrotoxicity
IP	1.0000	Nephrotoxicity
Eos	0.9759	
Mono	0.9759	
Neu	0.9789	
WBC	0.9819	
Bas	0.9849	
TG	0.9880	
PL	0.9910	
Ht	0.9910	
Plat	0.9910	
TC	0.9940	
MCH	0.9940	
PT	0.9940	
APTT	0.9940	
Fbg	0.9940	
GLC	0.9970	
Ret	0.9970	
TP	1.0000	
RALB	1.0000	
A/G	1.0000	
RBC	1.0000	
Hb	1.0000	
MCV	1.0000	
MCHC	1.0000	
Lym	1.0000	

Supplementary Table 1. The consistency between AnimalGAN results and real animal testing data on toxicity assessment for each of the 38 clinical pathology measurements. Two-tailed unpaired t-tests were performed to compare the controls and treatments, and $p < 0.05$ were considered statistically significant. Calculations were based on the hypothesis that the clinical pathology measurements are normally distributed and that the within-group variances are the same. For those measurements that did not follow the normal distribution, non-parametric tests were used. Then the consistency was calculated based on the comparison of toxicity assessment conclusions between AnimalGAN results and real testing data.

Generator G	Discriminator D
$\boldsymbol{z} \in \mathbb{R}^{\mathbf{1828}} \sim \boldsymbol{\mathcal{N}}(\mathbf{0}, \boldsymbol{I})$; $\boldsymbol{c} \in \mathbb{R}^{\mathbf{1828}}$	A sample $\boldsymbol{x} \in \mathbb{R}^{38}$ with condition $\boldsymbol{c} \in \mathbb{R}^{1828}$
$\boldsymbol{concat}(\boldsymbol{c}, \boldsymbol{z}) \in \mathbb{R}^{\mathbf{3656}}$	$concat(\boldsymbol{c}, \boldsymbol{x}) \in \mathbb{R}^{1866}$
Fully connected layer→4096; LeakyReLU	Fully connected layer→2048; LeakyReLU
Fully connected layer→2048; LeakyReLU	Fully connected layer→1024; LeakyReLU
Fully connected layer→1024; LeakyReLU	Fully connected layer→ 256; LeakyReLU
Fully connected layer→256; LeakyReLU	Fully connected layer→64; LeakyReLU
Fully connected layer→64; LeakyReLU	Fully connected layer→32; LeakyReLU
Fully connected layer→38	Fully connected layer→1

Supplementary Table 2. Network architectures for the generator and discriminator of AnimalGAN. The noise $\boldsymbol{z}$ is set to a vector of the same dimension as the condition $\boldsymbol{c}$ sampled from a multivariate Gaussian distribution $\boldsymbol{\mathcal{N}}(\mathbf{0}, \boldsymbol{I})$. The covariance matrix here is the identify matrix of size 1828, denoted by $\boldsymbol{I}$.

Supplementary Notes

Supplementary Note 1: Hyper-Parameter Tuning

To determine whether the model has achieved statistical convergence, we evaluate the cumulative loss of AnimalGAN across 1000 epochs. It's important to note that not all generated records are biologically valid. Hence, we calculate the ratio of valid records among all generated ones, referring to this as the "valid records ratio". This ratio serves as one criterion to demonstrate the model's biological relevance and performance. For the generated testing results, only those that pass the blood count check are used for the following analysis, and the invalid records are discarded.

Learning rate

We utilized the Adam optimizer to optimize the loss function, employing three key parameters: the initial learning rate, the decay rate of the first-order momentum of the gradient (denoted as beta1 or b1), and the decay rate of the second-order momentum of the gradient (denoted as beta2 or b2). Since our training dataset is relatively small, we used correspondingly small initial learning rates, b1 values and b2 values. In this step, we fixed other parameters to find a more suitable learning rate, b1 and b2 values. Ultimately, we selected an initial learning rate of 1e-7, a beta1 value of 0.8, and a beta2 value of 0.95 (**Supplementary Figure 7**). These values were then fixed for subsequent rounds of hyper-parameter tuning.

Batch size

We also conducted a search for an appropriate batch size. As a result of this search, a batch size of 128 was chosen for subsequent tuning steps, considering both model convergence and the valid records ratio (**Supplementary Figure 8**).

Number of discriminator Iterations

In each iteration, we train the generator once and the discriminator multiple times. We evaluated the influence of varying the number of discriminator iterations, considering values of 5, 10 and 20 (**Supplementary Figure 9**). Ultimately, we determined that using 5 iterations for the discriminator yield optimal results.

Noise length

A random noise is an input to the generator and the generator maps the noise distribution to the distribution of clinical pathology measurements. We also evaluated the impact of the noise length (the

dimension of the noise vector). Specifically, we tested noise lengths of 16, 38 (equal to the number of clinical pathology measurements), 64, 128 and 1828 (the dimension of the treatment conditions: descriptors + time + dose). **Supplementary Figure 10** indicated that the convergence and valid records ratio do not vary much across the various noise lengths. As a result, we opted to retain a noise length of 1828 for our model.

Supplementary Note 2: Using 2D molecular descriptors only to build model

In the main manuscript, we report the AnimalGAN, which utilizes both 2D and 3D molecular descriptors generated by Mordred. In addition, we also conducted a study using 2D molecular descriptors only. Similar to the reported AnimalGAN, we conducted parameter searches and model training, and the final model was employed to generate clinical pathology measurements for the same test dataset used in the reported AnimalGAN. The results from the model based on 2D descriptors demonstrated comparable performance to that of the AnimalGAN (**Supplementary Figure 11**).

Supplementary Note 3: Bland Altman analysis on 38 measurements

We did a Bland Altman analysis for all 38 measurements across all the treatment conditions, which was included in a **Supplementary Figure 12**. We did not observe a consistent bias of AnimalGAN over experimental results.

Supplementary Note 4: Evaluation based on MSEs across dose levels and time points

To comprehensively assess the performance of the AnimalGAN, we employed the mean squared error (MSE) as measure to compare the AnimalGAN-generated results against real experiment data for the treatment conditions in the test set. specifically, we focused on the following aspects:

Time Points:

We conducted an evaluation of the MSE over different time points after treatment (3, 7, 14, and 28 days), that is scarified period of 4, 8, 15 and 29 days. This analysis helps us ascertain that the model's performance is not driven by trivial optimizations that predominantly minimize the overall MSE by emphasizing specific time intervals. **Supplementary Figure 13 (A)** illustrates the MSE values across these time points.

Dose Level:

Additionally, we evaluated the MSE across various dose levels. This examination ensures that the model's performance is not skewed towards minimizing MSE by focusing solely on certain dosage levels. The corresponding **Supplementary Figure 13 (B)** provides a visualization of MSEs across different dose levels.

The depiction of MSE trends over time points and dose levels serves as compelling evidence that our model captures meaningful patterns and avoids overemphasizing specific components solely for the purpose of minimizing MSE.
