# Supplementary material for: A generative adversarial network model alternative to animal studies for clinical pathology assessment
Source: Nat Commun. 2023 Nov 6;14:7141. doi: 10.1038/s41467-023-42933-9 (PMC10628291; doi:10.1038/s41467-023-42933-9)
Supplement: Supplementary file 3 — Description of Additional Supplementary Files [file 41467_2023_42933_MOESM3_ESM.pdf]

File name: Supplementary Data 1

Description: The performance of AnimalGAN and 12 QSAR models for each of the 38 clinical pathology measurements.

File name: Supplementary Data 2

Description: The detailed information on treatment conditions and samples used in this study. Data splitting details in different scenarios are also include in this table.

File name: Supplementary Data 3

Description: **Compounds used in this study.** Compounds details, including PubChem CID, SMILES, structural similarity score calculated based on Mordred molecular representations, first level of the WHO Anatomical Therapeutical Chemical (ATC) code, and the initial approval year of each compound.

File name: Supplementary Data 4

Description: External validation dataset extracted from DrugMatrix.
